# Initiation of Methylglucose Lipopolysaccharide Biosynthesis in Mycobacteria

**DOI:** 10.1371/journal.pone.0005447

**Published:** 2009-05-07

**Authors:** Devinder Kaur, Ha Pham, Gérald Larrouy-Maumus, Michel Rivière, Varalakshmi Vissa, Marcelo E. Guerin, Germain Puzo, Patrick J. Brennan, Mary Jackson

**Affiliations:** 1 Mycobacteria Research Laboratories, Department of Microbiology, Immunology and Pathology, Colorado State University, Fort Collins, Colorado, United States of America; 2 Département «Mécanismes Moléculaires des Infections Mycobactériennes», Centre National de la Recherche Scientifique (CNRS), Institut de Pharmacologie et Biologie Structurale (UMR 5089), Université de Toulouse III, Toulouse, France; Baylor College of Medicine, United States of America

## Abstract

**Background:**

Mycobacteria produce two unique families of cytoplasmic polymethylated polysaccharides - the methylglucose lipopolysaccharides (MGLPs) and the methylmannose polysaccharides (MMPs) - the physiological functions of which are still poorly defined. Towards defining the roles of these polysaccharides in mycobacterial physiology, we generated knock-out mutations of genes in their putative biosynthetic pathways.

**Methodology/Principal Findings:**

We report here on the characterization of the Rv1208 protein of *Mycobacterium tuberculosis* and its ortholog in *Mycobacterium smegmatis* (MSMEG_5084) as the enzymes responsible for the transfer of the first glucose residue of MGLPs. Disruption of *MSMEG_5084* in *M. smegmatis* resulted in a dramatic decrease in MGLP synthesis directly attributable to the almost complete abolition of glucosyl-3-phosphoglycerate synthase activity in this strain. Synthesis of MGLPs in the mutant was restored upon complementation with wild-type copies of the *Rv1208* gene from *M. tuberculosis* or *MSMEG_5084* from *M. smegmatis*.

**Conclusions/Significance:**

This is the first evidence linking Rv1208 to MGLP biosynthesis. Thus, the first step in the initiation of MGLP biosynthesis in mycobacteria has been defined, and subsequent steps can be inferred.

## Introduction

**Figure 1:**
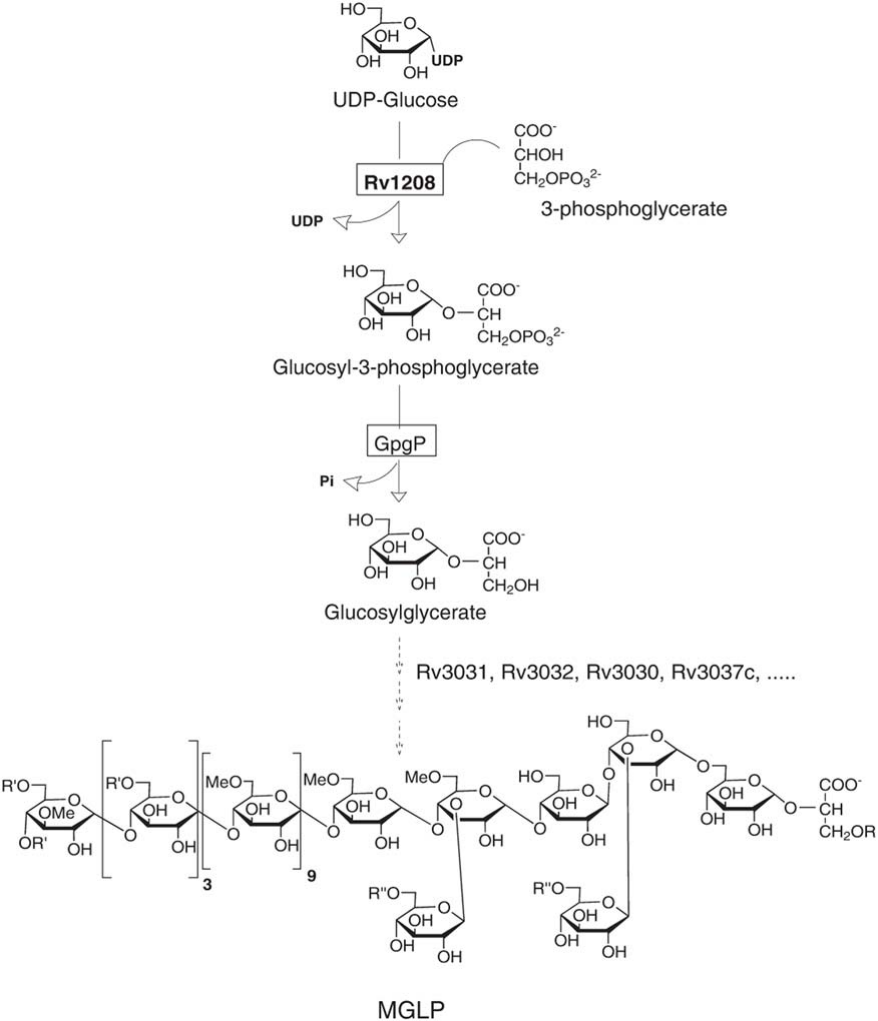
Structure and early biosynthetic steps of MGLPs. The catalytic steps leading to the synthesis of glucosylglycerate and genes thought to be involved in the elongation of MGLPs are represented. MGLPs from *M. bovis* BCG are composed of 10 α−(1→4)-linked 6-*O*-methylglucosyl residues with a non-reducing end made of the tetrasaccharide 3-*O*-methyl-D-Glc*p*-[α(1→4)-D-Glc*p*]_3_-α(1→. The tetrasaccharide →4)-[α−(1→4)-D-Glc*p*]_3_-α−(1→6)-D-Glc*p*-α−(1→linked to the position 2 of D-glyceric acid constitutes the reducing end of the molecule. The second and fourth α-D-Glc*p* residues (closest to the reducing end) are substituted at position 3 by single β-D-Glc*p* residues. The non-reducing end of the polymer is acylated by a combination of acetate, propionate and isobutyrate (R′), whereas octanoate (R) esterifies the position 1 of glyceric acid and zero to three succinate groups (R″) esterify the Glc residues of the reducing end. MGLPs occur as a mixture of four main components that differ in their content of esterified succinic acid.

Mycobacteria produce two cytoplasmic polymethylated polysaccharides (PMPS) of intermediate size in which many of the sugar units are partially *O*-methylated, thus conferring on the molecules a slight hydrophobicity [1]. One class is known as the 3-*O*-methylmannose polysaccharides (MMPs) [2]–[3] and the other as the 6-*O*-methylglucose lipopolysaccharides (MGLPs) (Fig. 1) [4]–[5]. The ability of both PMPS to form *in vitro* stable complexes with medium- and long-chain fatty acyl chains and acyl-CoAs and to regulate the activity of the fatty acid synthase I (FAS-I) has led to the hypothesis that these polysaccharides are important regulators of fatty acid and mycolic acid metabolism in mycobacteria [6]–[8]. These findings, however, have been derived from *in vitro* studies using enzyme assays and purified substrates and whether they accurately reflect the physiological function of PMPS in whole cells is not clear. With the ultimate goal of studying the physiological roles of MMPs and MGLPs, we have begun to define their biosynthetic pathways and to generate mycobacterial mutants deficient in different aspects of their biosynthesis [1], [9].

Our recent evidence indicates that two clusters of genes are likely to participate in the biosynthesis of MGLPs in *M. tuberculosis*. One encompasses *Rv3030-Rv3037c* in the genome of *M. tuberculosis* H37Rv [10] and carries the α-(1→4)-glucosyltransferase gene (*Rv3032*) responsible for the elongation of MGLPs [9], a putative acetyltransferase gene (*Rv3034c*), two putative SAM-dependent-methyltransferase genes (*Rv3030* and *Rv3037c*) and a potential branching gene (*Rv3031*) likely to be involved in the formation of the α-(1→6)-glycosidic bond linking the first and second D-Glc*p* residues at the reducing end of the molecule. The finding that a *M. tuberculosis* H37Rv knock-out mutant deficient in the Rv3032 enzyme still produced residual amounts of MGLPs then led us to identify Rv1212c as the likely compensatory α-(1→4)-glucosyltransferase [11]. Failure to disrupt both the *Rv3032* and *Rv1212c* genes in the same *M. tuberculosis* H37Rv strain further indicated that bacterial growth required at least one of these two genes to be functional. Whether this physiological requirement is particularly related to the synthesis of glycogen, capsular glucan or MGLPs has not yet been elucidated, since Rv3032 and Rv1212c appear to participate in the elongation of all three molecules and to partially compensate for one another [11]. Interestingly, *Rv1212c* also belongs to a cluster of genes (*Rv1208–Rv1213*) encoding putative sugar-modifying enzymes [1], [11]. The existence of a putative retaining glycosyltransferase of the recently established CAZy GT-81 family (http://www.cazy.org/), Rv1208, showing weak sequence similarities with the glucosyl-3-phosphoglycerate synthase (GpgS) from *Persephonella marina* (∼24% amino acid identity) in the vicinity of Rv1212c suggested that Rv1208 might catalyze the first glucosyl transfer in MGLP biosynthesis (Fig. 1) [12]. Supporting this assumption, recombinant forms of the orthologs of Rv1208 from *Mycobacterium bovis* BCG and *M. smegmatis* have been shown to display GpgS activity *in vitro* [13]. Moreover, the three-dimensional structures of Rv1208 and its ortholog in *M. avium* subsp. *paratuberculosis*, MAP2569c, in their apo forms and in complex with UDP, UDP-glucose, and both UDP and D-3-phosphoglycerate have been solved allowing the classification of these enzymes as GT-A-type glycosyltransferases and the molecular determinants for substrate recognition and catalysis to be established [14]–[15]. Direct evidence linking this enzyme to the biogenesis of MGLPs in mycobacteria was, however, lacking. *Rv1208* was predicted to be an essential gene of *M. tuberculosis* by high-density mutagenesis [16]. We thus undertook to analyze the effects of disrupting *MSMEG_5084*, the ortholog of *Rv1208* in *M. smegmatis*, on the GpgS activity and MGLP synthesis of this bacterium. An assay was developed which allowed the formation of the early precursors of MGLPs to be monitored in mycobacterial cell-free extracts for the first time.

## Results and Discussion

### Effects of knocking-out MSMEG_5084 on the biosynthesis of MGLP in M. smegmatis

**Figure 2:**
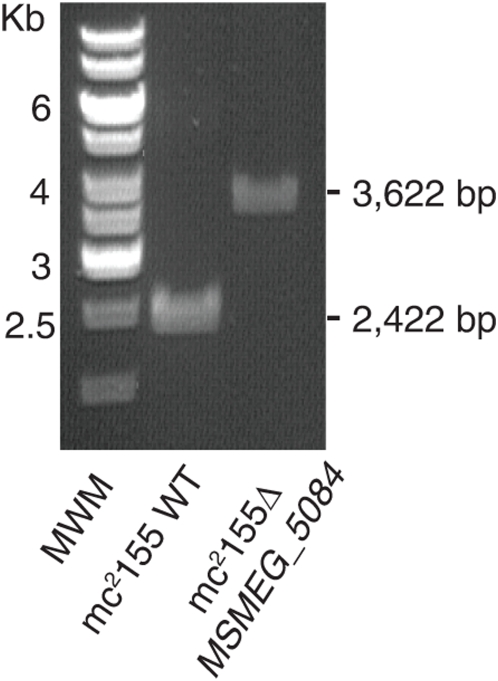
Generation of a *MSMEG_5084* knock-out mutant of *M. smegmatis*. Evidence for allelic replacement at the *MSMEG_5084* locus of *M. smegmatis*. Allelic replacement was confirmed by PCR using primers smeg1208.3 and smeg1208.4 (see Materials and Methods). The WT 2,422-bp amplification signal is replaced by a 3,622-bp fragment in the mutant due to the insertion of a 1.2 kb- kanamycin resistance cassette.

The ortholog of the *Rv1208* gene in *M. smegmatis* mc^2^155, *MSMEG_5084*, was disrupted by homologous recombination using standard protocols [17]. The product of *MSMEG_5084* (303 amino acids) shares 74% identity (84% similarity) with its *M. tuberculosis* counterpart (324 amino acids) on a 302 amino acid overlap. Allelic replacement at the *MSMEG_5084* locus was confirmed by PCR and Southern hybridization (Fig. 2 and data not shown). Complemented mutant strains were obtained by transforming mc^2^Δ*MSMEG_5084* either with pVV2*Rv1208*, expressing a wild-type copy of *Rv1208* from *M. tuberculosis* H37Rv or pVV16*MSMEG_5084*, expressing a wild-type copy of *MSMEG_5084* from *M. smegmatis* mc^2^155.

As compared to its wild-type parent *M. smegmatis* mc^2^155, the *MSMEG_5084* mutant displayed a significantly reduced growth rate at 37°C. Mutant colonies typically appeared 3 to 4 days later than wild-type colonies on 7H11-OADC, and 1 to 2 days later than mc^2^155/pVV16*MSMEG_5084* colonies. Thus, growth was partially restored in the complemented mutants.

**Figure 3:**
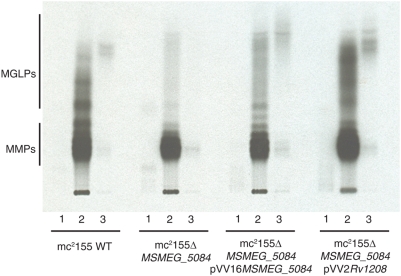
MGLP synthesis by *M. smegmatis* wild-type and *MSMEG_5084* mutant. TLC autoradiograph of the [^14^C-methyl]-L-methionine-labeled and partially purified MGLPs from mc^2^155 wild-type (WT), mc^2^Δ*MSMEG_5084*, mc^2^Δ*MSMEG_5084*/pVV2*Rv1208* and mc^2^Δ*MSMEG_5084*/pVV16*MSMEG_5084*. PMPS were extracted from bacterial cells and purified by reverse phase chromatography on SepPak® Plus tC18 columns (Waters); 1: fraction eluted with 20% methanol; 2: fraction eluted with 40% methanol; 3: fraction eluted with 70% methanol. MGLPs were prepared from the same amounts of wild-type, mutant and complemented mutant cells.

Analysis of the MGLPs produced by different culture batches of the wild-type and mutant strains metabolically labeled with [^14^C-methyl]-L-methionine [9] revealed a virtual elimination of the *de novo* production of these molecules in the mutant (Fig. 3). MGLP production was restored in the mutant complemented with wild-type copies of either *Rv1208* or *MSMEG_5084* indicating that the two genes are functional orthologs (Fig. 3). The total amount of radioactivity incorporated into the MGLPs of the mutant was only about 20% of that for the wild-type parent. MMP synthesis, in contrast, appeared relatively unaffected in mc^2^Δ*MSMEG_5084* indicating that a deficiency in MGLP production does not affect other PMPS (Fig. 3). These results clearly confirm the primary role of Rv1208 in the initiation of MGLP synthesis, however, since residual amounts of MGLPs were still produced in the mutant, we conclude that another enzyme displaying glucosyl-3-phosphoglycerate synthase or glucosylglycerate synthase activity contributed to the pool.

**Figure 4:**
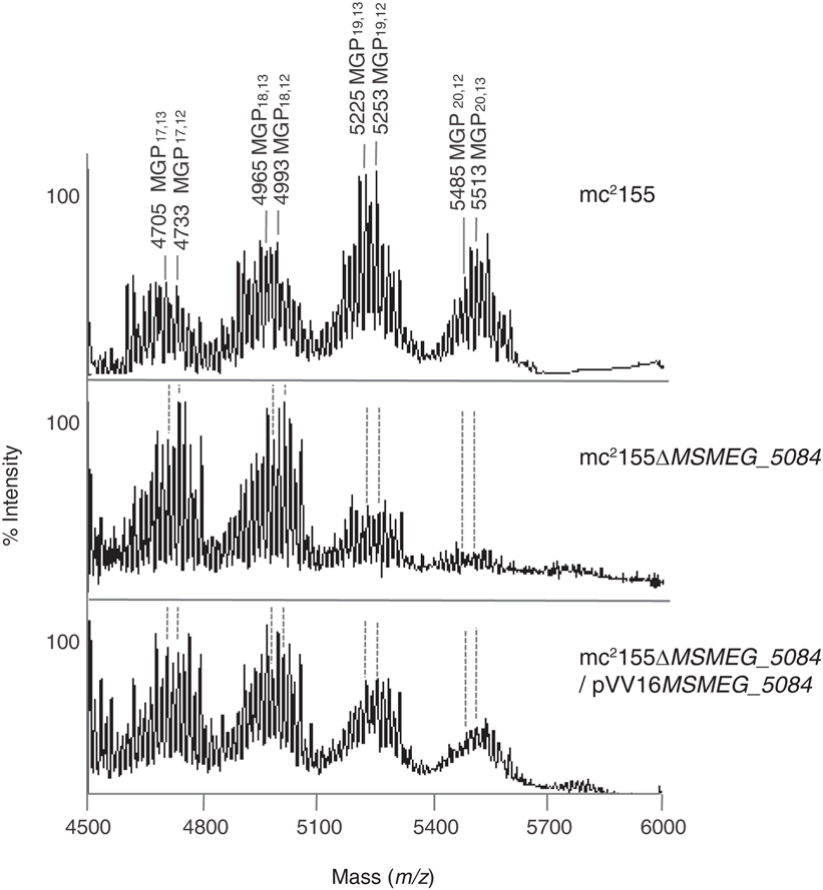
MGP composition of the wild-type, *MSMEG_5084* mutant and complemented mutant strains of *M. smegmatis*. Partial high mass range (*m/z* 4500–6000) MALDI-TOF mass spectra in the positive ion reflector mode of the purified MGPs (deacylated and peracetylated MGLPs) from *M. smegmatis* mc^2^155 wild-type, mc^2^Δ*MSMEG*_5084 and mc^2^Δ*MSMEG*_5084/pVV16*MSMEG*_5084. MGP_x,y_ denotes a form of MGP containing×glucosyl units of which y are *O*-methylated. All annotated peaks correspond to [M-H+2Na]^+^ pseudomolecular ions.

To compare the structures of the MGLPs produced by the wild-type, mutant and complemented mutant strains, they were purified by reverse phase chromatography, deacylated, peracetylated and analyzed by MALDI-TOF mass spectrometry. The mass spectrum of the MGP fraction of wild-type *M. smegmatis* mc^2^155 mainly showed four series of ions separated by 260 mass units and differing by 28 and 16 mass units attributable to the peracetylation of the samples (i.e., replacement of a methyl group by an acetyl group) and potassium adducts of the pseudomolecular ions, respectively (Fig. 4). The observed clustered ions thus reflect the variability of MGPs in terms of their degree of glycosylation, *O*-methylation and salt (sodium, potassium) adducts. In the wild-type strain, the two most intense ions at *m/z* 5253 and 5513 were assigned, respectively, to the [M-H+2Na]^+^ pseudomolecular ions of MGP_19,12_ and MGP_20,12_ (MGP containing 19 and 20 glucose units, among which 12 are *O*-methylated) (Fig. 4). In contrast, mc^2^Δ*MSMEG_5084* accumulated lower molecular weight MGP species, consisting of 17 and 18 Glc residues (MGP_17,12_, MGP_17,13_, MGP_18,12_, MGP_18,13_); although MGP_19,12_, MGP_19,13_, MGP_20,12_, MGP_20,13_ were detectable in the mutant strain, the abundance of these was clearly much less than in the wild-type strain (Fig. 4). Thus, disrupting *MSMEG*_5084 in *M. smegmatis* resulted primarily in simple, less polymerized structures, and also diminished yields. Complementation of the mutant strain with pVV16*MSMEG*_5084 partially restored the synthesis of the mature forms of MGPs (MGP_19,12_, MGP_20,12_) in the cells (Fig. 4).

No qualitative or quantitative differences were found between the mutant and wild-type strains in terms of fatty acid and mycolic acid contents (Fig. S1). Thus, altered biosynthetic patterns of MGLPs had no significant impact on fatty acid metabolism. This result is consistent with observations reported by Dr. Ballou's laboratory on a spontaneous mutant of *M. smegmatis* with defects in MMP and MGLP synthesis [18] and our own observations on a *Rv3032* knock-out mutant of *M. tuberculosis* H37Rv and a *MSMEG_2350* knock-out mutant of *M. smegmatis* mc^2^155, both of which were found to be significantly impaired in MGLP biosynthesis but not in that of fatty acids and mycolates [9]. Although the presence of MMPs (which are thought to play similar regulatory functions as MGLPs on fatty acid synthesis) in mc^2^Δ*MSMEG_5084* might account at least in part for this result, this study casts further doubts on the prevailing hypothesis that MGLPs are, *in vivo*, involved in the regulation of fatty acid synthesis [1].

### Effects of knocking-out MSMEG_5084 on the biosynthesis of MGLP precursors by M. smegmatis cell-free extracts

**Figure 5:**
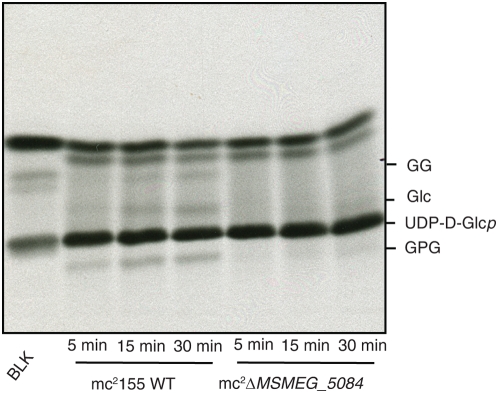
Effects of knocking-out *MSMEG_5084* on the biosynthesis of MGLP precursors by *M. smegmatis* cell-free extracts. Radiolabeled assays using *M. smegmatis* wild-type (mc^2^155 WT) and mutant (mc^2^Δ*MSMEG_5084*) extracts. Reaction products were analyzed by TLC as described under Materials and Methods and revealed by autoradiography. Incubation times are indicated. BLK, blank (no bacterial lysates); GG, glucosylglycerate; GPG, glucosyl-3-phosphoglycerate; Glc, glucose.

To directly correlate *Rv1208* and glucosyl-3-phosphoglycerate synthase activity to the biosynthesis of MGLPs in mycobacterial cells, whole cell lysates prepared from the wild-type and mutant strains of *M. smegmatis* provided enzyme sources in assays aimed at monitoring the formation of glucosylglycerate and MGLP precursors *in vitro*. UDP-D-[U-^14^C]Glc and D-3-phosphoglycerate served as the donor and acceptor substrates, respectively. Time-dependent formation of glucosyl-3-phosphoglycerate (GPG) was clearly visible in the cell-free extracts of wild-type *M. smegmatis* (Fig. 5). As expected, this product was progressively dephosphorylated by an unknown endogenous phosphatase, GlgP (Fig. 1), to yield glucosylglycerate (GG) (Fig. 5). In contrast, barely detectable amounts of GPG and no GG were detected in the assays using mc^2^Δ*MSMEG_5084* extracts (Fig. 5), even after prolonged incubation times.

In conclusion, the disruption of *MSMEG_5084* in *M. smegmatis* results in an 80% decrease in the production of MGLPs directly attributable to a drastic if not complete loss of GPG synthesis. The dispensability of the *MSMEG_5084* gene for the growth of *M. smegmatis* while its ortholog in *M. tuberculosis* H37Rv, *Rv1208*, is predicted to be essential may be accounted for by the existence of residual amounts of MGLPs in mc^2^Δ*MSMEG_5084* or by the production by *M. smegmatis* mc^2^155 but not *M. tuberculosis* H37Rv of MMPs thought to display the same physiological functions as MGLPs [1]. Altogether, our results indicate that *Rv1208* and its ortholog in *M. smegmatis* encode glucosyl-3-phosphoglycerate synthases involved in the transfer of the first glucosyl residue of MGLPs. Results also confirm the participation of two gene clusters in the biosynthesis of these lipopolysaccharides in the genomes of mycobacteria. The existence of endogenous enzymes with weak glucosyl-3-phosphoglycerate synthase or glucosylglycerate synthase activity probably account for the residual synthesis of MGLPs in the *MSMEG_5084* mutant of *M. smegmatis*.

## Materials and Methods

### Construction of a M. smegmatis MSMEG_5084 knock-out mutant

A two-step homologous recombination procedure using the counterselectable marker *sacB* [17] was used to achieve allelic replacement at the *MSMEG_5084* locus of *M. smegmatis* mc^2^155. The *MSMEG_5084* gene and flanking regions were PCR-amplified from *M. smegmatis* mc^2^155 genomic DNA using the forward (smeg1208.1) 5′-tataagaattcctacggcatcagcgagcg-3′ and reverse (smeg1208.2) 5′-tataagcggccgcgcggttgcgcacgatcc-3′ primers and a disrupted allele, *MSMEG_5084::km*, was obtained by inserting the kanamycin resistance cassette from pUC4K (Amersham Pharmacia Biotech) into the PstI restriction site of *MSMEG_5084*. *MSMEG_5084::km* was then cloned into the NotI-cut and blunt-ended pJQ200-*xylE* [17] to obtain pJQ200-xylE- *MSMEG_5084::km*, the construct used for allelic replacement. Allelic replacement at the *MSMEG_5084* locus was confirmed by PCR using primers smeg1208.3 (5′-atcgagtggctgcgcagc-3′) and smeg1208.4 (5′-gcacttgcgacatgtcgg-3′) and Southern blot as described [19].

For complementation studies, the entire coding sequence of *Rv1208* was PCR-amplified from *M. tuberculosis* H37Rv genomic DNA using the primers 5′-tataacatatgacagcatcggagctggtc-3′ and 5′-tataaaagcttcagcgcggccgcatcac-3′ and cloned into the NdeI and HindIII restriction sites of the expression vector pVV2, yielding pVV2*Rv1208*. pVV2 is a shuttle *E. coli/Mycobacterium* plasmid derived from pMV261 [20]. It harbors kanamycin and hygromycin-resistance markers and allows the constitutive production of N-terminal His_6_-tagged proteins in mycobacteria under the control of the p*hsp60* promoter. *MSMEG_5084* was PCR-amplified using the primers smeg1208.1 and smg1208.2 described above and cloned into the blunted *SpeI* site of pVV16 [19] for expression from its own promoter.

### Whole cell radiolabeling experiments

Radiolabeling with [^14^C-methyl]-L-methionine (0.5 µCi ml^−1^; specific activity, 55 Ci mol^−1^, Amersham) was performed at 37°C in Sauton's medium for 24 hr as described [9].

### Purification and analysis of MGLPs

MGLPs were extracted and purified from cold and radiolabeled *M. smegmatis* Sauton's cultures as previously described [9]. TLC analyses were performed on aluminum-backed silica gel 60-precoated plates F_254_ (E. Merck, Darmstadt, Germany) using chloroform/methanol/water (56∶38∶10, by vol.) as the eluent. Radiolabeled MGLPs were visualized by exposure of TLC plates to Kodak X-Omat AR films at −80°C. MGPs were obtained by deacylating dry MGLPs in 1 M NaOH at 37°C for 3 hr as described [9]. Samples (5–10 µg) were peracetylated with 100 µl acetic anhydride in the presence of 100 µl of pyridine for 1 hr at 110°C. After removal of the solvent, peracetylated MGPs were extracted three times with chloroform/water (1∶1). The chloroform phases were pooled, washed three times with water, and the dried residue dissolved in 10 µl chloroform prior to Matrix-Assisted Laser Desorption-Ionization Time-Of-Flight (MALDI-TOF) Mass Spectrometry (MS). Typically, 0.5 µl of the peracetylated MGP sample in chloroform was mixed with 0.5 µl of matrix solution (dihydroxybenzoic acid 10 mg/ml in 50% ethanol) directly on the MALDI target plate. MALDI-TOF MS analyses were performed on a MALDI-TOF Voyager DE STR (Applied Biosystems, Framinham, MA) calibrated using the instrument's external calibration procedure, in positive ion reflector mode using 20 kV accelerating voltage with an extraction delay of 200 nsec.

### Glucosyl-3-phosphoglycerate synthase assays

Enzymatically-active whole cell-free extracts from *M. smegmatis* were prepared by sonication of *M. smegmatis* cells for 8 min under the form of 8×60 s-pulses with 90 s-cooling intervals between pulses followed by centrifugation of the extracts at 15,000×*g* for 30 min to remove unbroken cells. Assays performed with mycobacterial cell-free extracts contained 0.5 mM (0.05 µCi) UDP-D-[U-^14^C]Glc (GE Healthcare), 1 to 3 mM D-3-phosphoglycerate (Sigma), 20 mM MgCl_2_, 150 to 300 µg of *M. smegmatis* proteins and 25 mM Tris-HCl buffer pH 8.0 in a total volume of 100 µl. Reaction mixtures were incubated at 37°C for 5 to 30 min and terminated by cooling in ethanol/dry ice. Reaction products were separated by TLC on aluminum-backed silica gel 60-precoated plates F_254_ developed in 1-propanol/ethyl acetate/water/25% ammonia (50∶10∶30∶10 by vol.) and revealed by autoradiography. The products of the reactions were characterized by co-migration with authentic glucosylglycerate (GG) and glucosyl-3phosphoglycerate (GPG) standards produced *in vitro* by *M. smegmatis* mc^2^155 cell free extracts. GPG was purified by preparative TLC and structurally characterized using ElectroSpray Ionisation mass spectrometry (ESI/MS) on a 6220 TOF (Agilent Technologies) in the negative ion mode (Fig. S2). GG was produced by treating purified GPG with 4 U alkaline phosphatase (Sigma) for 30 min at 37°C.

### Fatty acid and mycolic acid analysis

Fatty acid and mycolic acid methyl esters were prepared from whole *M. smegmatis* cells by methanolysis using methanolic-HCl (Supelco). Mycolic acid methyl esters were analyzed by TLC using *n*-hexane/ethyl acetate (95∶5; three developments) as the eluent. Fatty acid methyl esters were analyzed by gas chromatography-mass spectrometry (GC-MS) on a Varian CP-3800 gas chromatograph equipped with a Varian 320-MS TQ mass spectrometer using a 5% phenyl-methyl low bleed Factor Four GC column operating at a temperature of 50°C for 1 min followed by programmed increases of 30°C per min to 100°C and 10°C per min to 300°C.

## Supporting Information

Figure S1 Comparative analysis of the fatty acid and mycolic acid compositions of wild-type M. smegmatis mc2155 and mc2(delta)MSMEG_5084. Wild-type mc2155 and mc2(delta)MSMEG_5084 were grown in Sauton's medium as surface pellicles at 37°C. A) Mycolic acid methyl esters (MAMEs) were analyzed by TLC using n-hexane/ethyl acetate (95:5; three developments) as the eluent and revealed by charring with cupric sulfate (10% in a 8% phosphoric acid solution); B) Fatty acid methyl esters (FAMEs) were analyzed by gas chromatography-mass spectrometry. Shown are the relative percentages of each fatty acid in the strains.
(0.03 MB PDF)

Figure S2 Negative ion ESI/MS of GPG. GPG was purified by preparative TLC. The spectrum in the m/z range 335–385 atomic mass units (amu) is shown.
(0.02 MB PDF)
