# Supplementary material for: Initiation of Methylglucose Lipopolysaccharide Biosynthesis in Mycobacteria
Source: PLoS One. 2009 May 7;4(5):e5447. doi: 10.1371/journal.pone.0005447 (PMC2674218; doi:10.1371/journal.pone.0005447)
Supplement: Figure S1 — Comparative analysis of the fatty acid and mycolic acid compositions of wild-type M. smegmatis mc2155 and mc2(delta)MSMEG_5084. Wild-type mc2155 and mc2(delta)MSMEG_5084 were grown in Sauton's medium as surface pellicles at 37°C. A) Mycolic acid methyl esters (MAMEs) were analyzed by TLC using n-hexane/ethyl acetate (95:5; three developments) as the eluent and revealed by charring with cupric sulfate (10% in a 8% phosphoric acid solution); B) Fatty acid methyl esters (FAMEs) were analyzed by gas chromatography-mass spectrometry. Shown are the relative percentages of each fatty acid in the strains. (0.03 MB PDF) [file pone.0005447.s001.pdf]

Figure S1: Comparative analysis of the fatty acid and mycolic acid compositions of wild-type *M. smegmatis* $mc^2155$ and $mc^2\Delta$*MSMEG_5084*.

Wild-type $mc^2155$ and $mc^2\Delta$*MSMEG_5084* were grown in Sauton's medium as surface pellicles at 37°C. A) Mycolic acid methyl esters (MAMEs) were analyzed by TLC using *n*-hexane/ethyl acetate (95:5; three developments) as the eluent and revealed by charring with cupric sulfate (10% in a 8% phosphoric acid solution); B) Fatty acid methyl esters (FAMEs) were analyzed by gas chromatography-mass spectrometry. Shown are the relative percentages of each fatty acid in the strains.

A)

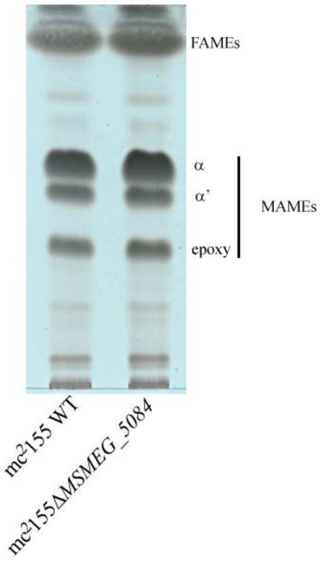


B)

| Strain | Fatty acid | | | | | | | |
|---|---|---|---|---|---|---|---|---|
| | C16:1 | C16:0 | C18:1 | C18:0 | C19 | C22:0 | C24:0 | C26:0 |
| $mc^2155$ WT | 10.10 | 29.41 | 19.45 | 7.93 | 14.58 | 3.73 | 14.16 | 0.59 |
| $mc^2\Delta$*MSMEG_5084* | 10.52 | 28.73 | 20.56 | 6.38 | 17.94 | 3.46 | 11.79 | 0.58 |
