# Supplementary material for: Initiation of Methylglucose Lipopolysaccharide Biosynthesis in Mycobacteria
Source: PLoS One. 2009 May 7;4(5):e5447. doi: 10.1371/journal.pone.0005447 (PMC2674218; doi:10.1371/journal.pone.0005447)

Figure S2: Negative ion ESI/MS of GPG.
GPG was purified by preparative TLC. The spectrum in the *m/z* range 335-385 atomic mass units (*amu*) is shown.

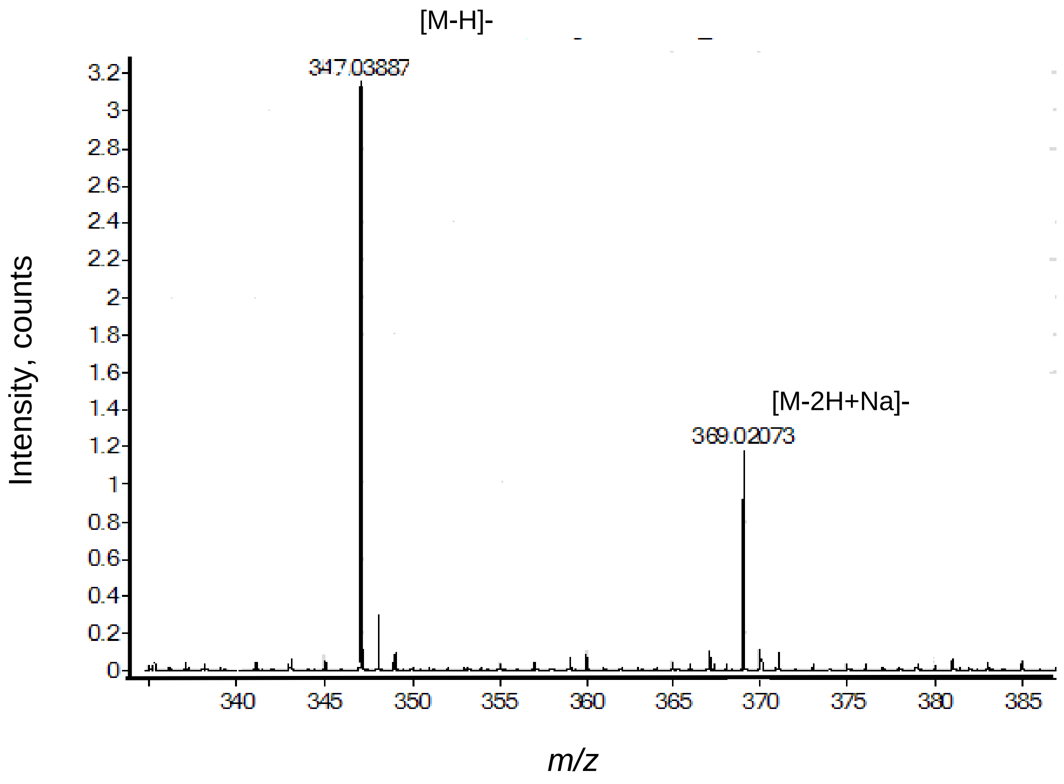

Supplement: Figure S2 — Negative ion ESI/MS of GPG. GPG was purified by preparative TLC. The spectrum in the m/z range 335–385 atomic mass units (amu) is shown. (0.02 MB PDF) [file pone.0005447.s002.pdf]
